# The novel μ‐opioid receptor agonist PZM21 depresses respiration and induces tolerance to antinociception

**DOI:** 10.1111/bph.14224

**Published:** 2018-05-14

**Authors:** Rob Hill, Alex Disney, Alex Conibear, Katy Sutcliffe, William Dewey, Stephen Husbands, Chris Bailey, Eamonn Kelly, Graeme Henderson

**Affiliations:** ^1^ School of Physiology, Pharmacology and Neuroscience University of Bristol Bristol UK; ^2^ Department of Pharmacy and Pharmacology University of Bath Bath UK; ^3^ Department of Pharmacology and Toxicology Virginia Commonwealth University Richmond VA USA

## Abstract

**Background and Purpose:**

PZM21 is a novel μ‐opioid receptor ligand that has been reported to induce minimal arrestin recruitment and be devoid of the respiratory depressant effects characteristic of classical μ receptor ligands such as morphine. We have re‐examined the signalling profile of PZM21 and its ability to depress respiration.

**Experimental Approach:**

G protein (G_i_) activation and arrestin‐3 translocation were measured *in vitro,* using BRET assays, in HEK 293 cells expressing μ receptors. Respiration (rate and tidal volume) was measured in awake, freely moving mice by whole‐body plethysmography, and antinociception was measured by the hot plate test.

**Key Results:**

PZM21 (10^−9^ – 3 × 10^−5^ M) produced concentration‐dependent G_i_ activation and arrestin‐3 translocation. Comparison with responses evoked by morphine and DAMGO revealed that PZM21 was a low efficacy agonist in both signalling assays. PZM21 (10–80 mg·kg^−1^) depressed respiration in a dose‐dependent manner. The respiratory depression was due to a decrease in the rate of breathing not a decrease in tidal volume. On repeated daily administration of PZM21 (twice daily doses of 40 mg·kg^−1^), complete tolerance developed to the antinociceptive effect of PZM21 over 3 days but no tolerance developed to its respiratory depressant effect.

**Conclusion and Implications:**

These data demonstrate that PZM21 is a low efficacy μ receptor agonist for both G protein and arrestin signalling. Contrary to a previous report, PZM21 depresses respiration in a manner similar to morphine, the classical opioid receptor agonist.

**Abbreviations**
DAMGO: D‐Ala^2^, N‐MePhe^4^, Gly‐ol‐enkephalin
GIRK: G protein‐activated inwardly rectifying potassium channel
MPE: maximum possible effect
MV: minute volume
PZM21: 1‐[(2S)‐2‐(dimethylamino)‐3‐(4‐hydroxyphenyl)propyl]‐3‐[(2S)‐1‐(thiophen‐3‐yl)propan‐2‐yl]urea
Rluc: *Renilla* luciferase

## Introduction

Opioid‐induced respiratory depression is the primary cause of death following illicit or prescription opioid misuse. Fatal opioid overdose accounted for over 140 deaths a day in the USA in 2016 (National Institutes of Health). In a significant proportion of these deaths, a prescription opioid or illicitly synthesized opioid, rather than http://www.guidetopharmacology.org/GRAC/LigandDisplayForward?ligandId=9082, was involved. The development of an opioid agonist with appreciable potency as an analgesic, but lacking the ability to induce respiratory depression, would be highly desirable for clinical application and to reduce opioid‐related deaths.

Current therapeutically useful opioid ligands such as http://www.guidetopharmacology.org/GRAC/LigandDisplayForward?ligandId=1627 produce their analgesic and respiratory depressant effects through activation of the http://www.guidetopharmacology.org/GRAC/ObjectDisplayForward?objectId=319 (Matthes *et al.,* 1996). The μ receptor is a GPCR that signals through activation of G_i_/G_o_ proteins and through arrestin signalling (Williams *et al.,* 2013). In recent years, the concept of biased ligands for GPCRs has been developed (Kelly, 2013; Kenakin and Christopoulos, 2013; Thompson *et al.,* 2015; Smith *et al.,* 2018). It is now well established that agonists acting at the same orthosteric site on a GPCR may activate different signalling pathways, that is, be biased for activating G protein or arrestin signalling. The idea that a G protein‐biased μ receptor agonist would have a reduced ‘on target’ side effect profile arises from the observation that in arrestin‐3 (β‐arrestin 2) knockout mice, http://www.guidetopharmacology.org/GRAC/LigandDisplayForward?ligandId=1627, the prototypical opioid agonist, showed a reduced propensity to produce respiratory depression and constipation (Raehal *et al.,* 2005) implying that respiratory depression resulted from μ receptor coupling to arrestin‐mediated signalling.

Manglik *et al*. (2016) reported the development of a novel μ receptor agonist, http://www.guidetopharmacology.org/GRAC/LigandDisplayForward?ligandId=9286, structurally unrelated to prototypical μ receptor agonists such as morphine, which appeared to have some selectivity for G protein activation over arrestin translocation. They also reported that PZM21 had a greatly reduced propensity to produce respiratory depression. We have synthesized PZM21 and have re‐examined its selectivity profile for G_i_ activation over arrestin‐3 recruitment and its ability to depress respiration in mice. We observed that PZM21 was a low efficacy agonist for both G_i_ activation and arrestin recruitment. In contrast to Manglik *et al*. (2016), we observed that PZM21 significantly depressed respiration, in a manner similar to an equi‐antinociceptive dose of morphine.

## Methods

#### G_i_ activation and arrestin‐3 translocation assays

HEK 293 cells were cultured at 37°C in DMEM supplemented with 10% fetal bovine serum and penicillin/streptomycin. Cells were seeded onto 10 cm dishes and grown to 80% confluence before transfection. To determine the relative ability of the agonists to activate Gα_i_ G proteins, a BRET^2^‐based assay that monitors the separation of Gα_i1_ and Gγ_2_ was used. HEK 293 cells were transiently transfected with rat HA‐μ receptors, Gα_i1_‐*Renilla* luciferase II (RlucII) and GFP10‐Gγ_2_. Immediately prior to each assay, cells were resuspended in clear DMEM and then transferred to a 96‐well plate at 90 μL per well. Measurements of BRET were made at 37°C. Coelenterazine 400a, at a final concentration of 5 μM, was injected 5 s prior to reading the cell plate. BRET measurements were made on a FLUOstar Omega plate reader (BMG LABTECH, Ortenberg, Germany) using the following filter set: acceptor, 515 ± 30 nm; and donor, 410 ± 80 nm filters. BRET signals were determined as the ratio of the light emitted by acceptors (GFP10) over donor (RlucII). For G_i_ activation, BRET measurements were taken 2 min after agonist application. Agonist stimulation resulted in a rapid decrease in the BRET signal between Gα_i1_‐RlucII and GFP10‐Gγ_2_ in cells co‐expressing the HA‐tagged μ receptors. To determine the extent of agonist‐induced arrestin‐3 association, cells were co‐transfected with human μ receptor‐Rluc and arrestin‐3‐GFP. The extent of arrestin‐3 recruitment to the receptor was assessed following a 10 min incubation with the agonists.

#### Animals

All animal care and experimental procedures were performed in accordance with the UK Animals (Scientific Procedures) Act 1986, the European Communities Council Directive (2010/63/EU) and the University of Bristol ethical review document. Animal studies are reported in compliance with the ARRIVE guidelines (Kilkenny *et al*., 2010; McGrath and Lilley, 2015). Male C57BL and CD‐1 mice (Harlan Laboratories, Bicester, UK) weighing approximately 30 g were group housed, four to six per cage, in an environment maintained at 22°C, on a reversed 12 h dark–light cycle with food and water available *ad libitum*. Experiments were performed in the dark phase to ensure that they were in their most active phase. Animals were randomly ascribed to treatment groups with the experimenter blinded to drug treatment until after subsequent data analyses had been performed. A total of 180 mice were used in the study.

#### Mouse respiration

We have used both C57BL mice, the strain used by Manglik *et al*. (2016), and CD‐1 mice, the strain we have used in previous studies of opioid depression of respiration (Hill *et al.,* 2016; Lyndon *et al.,* 2017; Withey *et al.,* 2017) to ensure that any responses observed were not strain‐specific. Respiration was measured in freely moving mice using plethysmography chambers (EMKA Technologies, Paris, France) supplied with either air or a 5% CO_2_ in air mixture (BOC Gas Supplies, Manchester, UK) as described previously (Hill *et al.,* 2016). Rate and volume of respiration were recorded and averaged over 5 min periods. Breathing 5% CO_2_ in air increases minute volume (MV) but does not induce stress in mice (Hill *et al.,* 2016).

Data are presented both as MV and as percentage change from the pre‐drug MV baseline, calculated for each mouse individually before mean data were plotted. Presenting data as percentage change from the pre‐drug levels has been done to control for variation between treatment groups that may have different baseline levels of respiration. In our experience, variations in baseline respiration levels do not influence the extent of opioid depression of respiration (Hill and Henderson, unpublished data).

#### Measurement of nociception

Male mice (CD‐1) were placed on a hot plate at 52.5°C, and the latency to exhibit paw withdrawal, jumping or paw licking was measured. A maximum cut‐off time of 30 s was used to prevent tissue damage. Hot plate latency was measured every 15 min. Antinociception was quantified as the percentage of maximum possible effect (% MPE), which was calculated as follows:

$$\%\mathrm{MPE}=\left[\left(\text{test latency}-\text{control latency}\right)/\left(30-\text{control latency}\right)\right]\times 100.$$

#### Induction of tolerance

Male mice (CD‐1) received twice daily injections (at 09:00 and 21:00 h each day) of PZM21, morphine or saline at 12 h intervals for 5 days. Two separate sets of experiments were performed, one to measure tolerance to antinociception and the other to measure tolerance to respiratory depression. Each day, hot plate latency or respiration was monitored for 60 min after the first drug administration of the day.

### Experimental design and data analysis

#### In vitro experiments

The maximum responses for PZM21, http://www.guidetopharmacology.org/GRAC/LigandDisplayForward?ligandId=1647 and morphine in both G_i_ activation and arrestin‐3 recruitment were compared using one‐way ANOVA with Bonferroni's post test.

#### In vivo experiments

Data from previous experiments where respiratory depression or antinociception was measured following acute opioid administration in naïve mice were subjected to *post hoc* power analyses using G*Power (version 3.1.9). Our calculations indicated that *n* = 6 (respiration experiments) or *n* = 8 (antinociception experiments) for each individual group would produce a significant result if an actual effect occurred.

Differences between drug treatments and saline controls over time were analysed using two‐way ANOVA with Bonferroni's post test. Changes in groups over time with repeat measurements were analysed using two‐way repeated measures ANOVA with Bonferroni's post test to analyse drug effect over time. Statistical significance and F (dfn, dfd) values are given in the figure legends. GraphPad Prism 7 was used for all statistical analyses. All data are displayed as mean ± SEM. The data and statistical analyses comply with the recommendations on experimental design and analysis in pharmacology (Curtis *et al.,* 2015).

#### Materials

PZM21 hydrochloride was synthesized from enantiopure (*S*)‐1‐(thiophen‐3‐yl)propan‐2‐amine and enantiopure (*S*)‐4‐(3‐amino‐2‐(dimethylamino)propyl)phenol (both supplied by Astatech, Bristol, Pennsylvania). The synthesis followed the methods reported by Manglik *et al*. (2016). Analysis by NMR, mass spectrometry and HPLC confirmed identity and purity of the sample. Reverse phase HPLC (Kinetix C18 column) and chiral analysis (Chiralpak AS column) demonstrated it to be identical to a sample of *S,S* PZM21 formate kindly provided by Dr Gmeiner (Friedrich‐Alexander‐Universität, Erlangen‐Nürnberg, Germany) and confirmed >98% of the desired isomer (*S*,*S* PZM21). Data using both batches of PZM21 (i.e. those synthesized at the University of Bath and at Friedrich‐Alexander‐Universität) are reported in this paper.

DAMGO (Tocris Bioscience, Avonmouth, UK), morphine hydrochloride (Macfarlan Smith, Edinburgh, UK), http://www.guidetopharmacology.org/GRAC/LigandDisplayForward?ligandId=1638 hydrochloride (Sigma‐Aldrich, Dorset, UK) and PZM21 hydrochloride were dissolved in deionized water (*in vitro* experiments) or sterile saline (*in vivo* experiments). PZM21 formate was dissolved in saline/1% DMSO. All doses of drug were administered in 0.1 mL vehicle.

### Nomenclature of targets and ligands

Key protein targets and ligands in this article are hyperlinked to corresponding entries in http://www.guidetopharmacology.org, the common portal for data from the IUPHAR/BPS Guide to PHARMACOLOGY (Harding *et al.,* 2018), and are permanently archived in the Concise Guide to PHARMACOLOGY 2017/18 (Alexander *et al.,* 2017).

## Results

### μ receptor signalling

**Figure 1:**
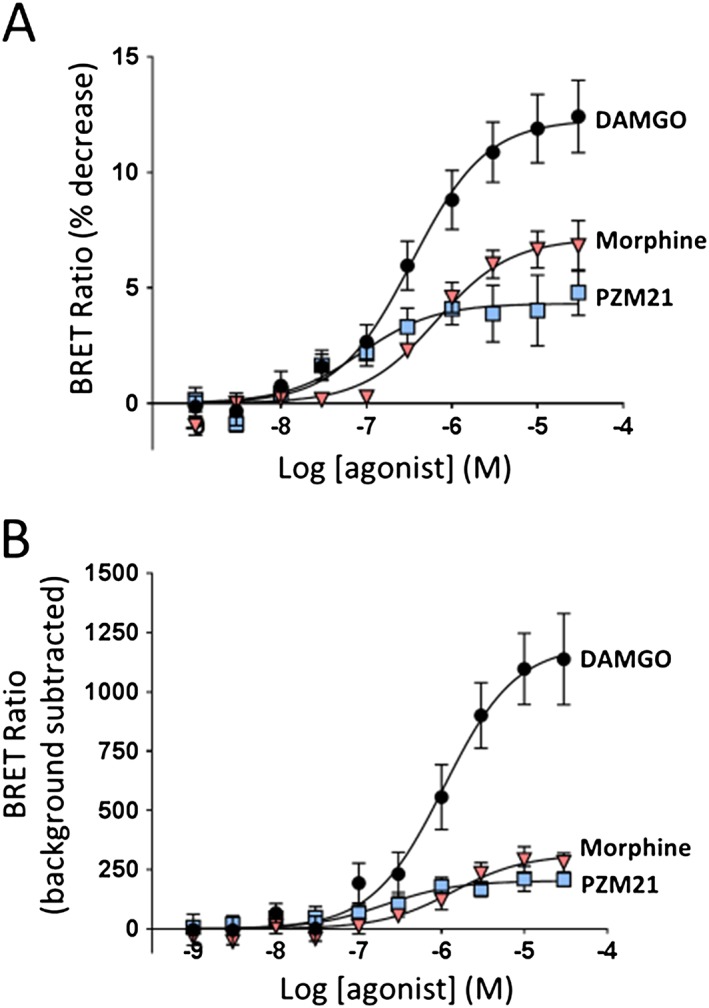
Opioid‐induced G_i_ protein activation and arrestin‐3 translocation in HEK 293 cells expressing recombinant μ receptors. (A) DAMGO, morphine and PZM21 induced concentration‐dependent activation of G_i_ measured as the decrease in BRET signal. (B) DAMGO, morphine and PZM21 induced concentration‐dependent arrestin‐3 translocation measured as the increase in BRET signal. Values of EC_50_ and maximum response are recorded in Table 1. Data shown are means ± SEM, *N* = 5 for each drug in both assays.

In HEK 293 cells expressing μ receptors, PZM21 (10^−9^ – 3 × 10^−5^ M) produced concentration‐dependent G_i_ activation (Figure 1A and Table 1). We compared the responses evoked by PZM21 to those evoked by two well‐characterized μ receptor ligands, DAMGO (high efficacy agonist) and morphine (lower efficacy agonist) in the same assay. Examination of the maximum response produced by each agonist (an indication of agonist efficacy) (Table 1) revealed that under our assay conditions, PZM21 and morphine were partial agonists for G_i_ activation with significantly lower efficacy than DAMGO.

**Table 1:** EC_50_ and maximum response values for DAMGO, morphine and PZM21 in the G_i_ coupling and arrestin‐3 recruitment assays

	G_i_ coupling			Arrestin‐3 recruitment		
Agonist	DAMGO	Morphine	PZM21	DAMGO	Morphine	PZM21
EC_50_ (M)	3.9 × 10^−7^ ± 0.7 × 10^−7^	6.6 × 10^−7^ ± 0.9 × 10^−7^	1.1 × 10^−7^ ± 0.4 × 10^−7^	1.2 × 10^−6^ ± 0.3 × 10^−6^	2.3 × 10^−6^ ± 0.9 × 10^−6^	4.5 × 10^−7^ ± 2.4 × 10^−7^
Maximum response (BRET ratio)	12.4 ± 1.6	6.8 ± 1.1*	4.8 ± 1.0*	1139 ± 193	281 ± 40*	208 ± 29*
Maximum response (% of DAMGO)	100	55	39	100	25	18

* *P* < 0.05, significantly different from the respective DAMGO value. The maximum response to PZM21 was not statistically different from that for morphine for either G_i_ coupling or arrestin‐3 recruitment.

In the arrestin‐3 recruitment assay PZM21 (10^−9^ – 3 × 10^−5^ M) produced a small but measurable amount of arrestin recruitment (Figure 1B). The maximum responses to PZM21 and morphine were lower than that of DAMGO whereas PZM21 and morphine were not significantly different.

### Acute antinociception and respiratory depression

**Figure 2:**
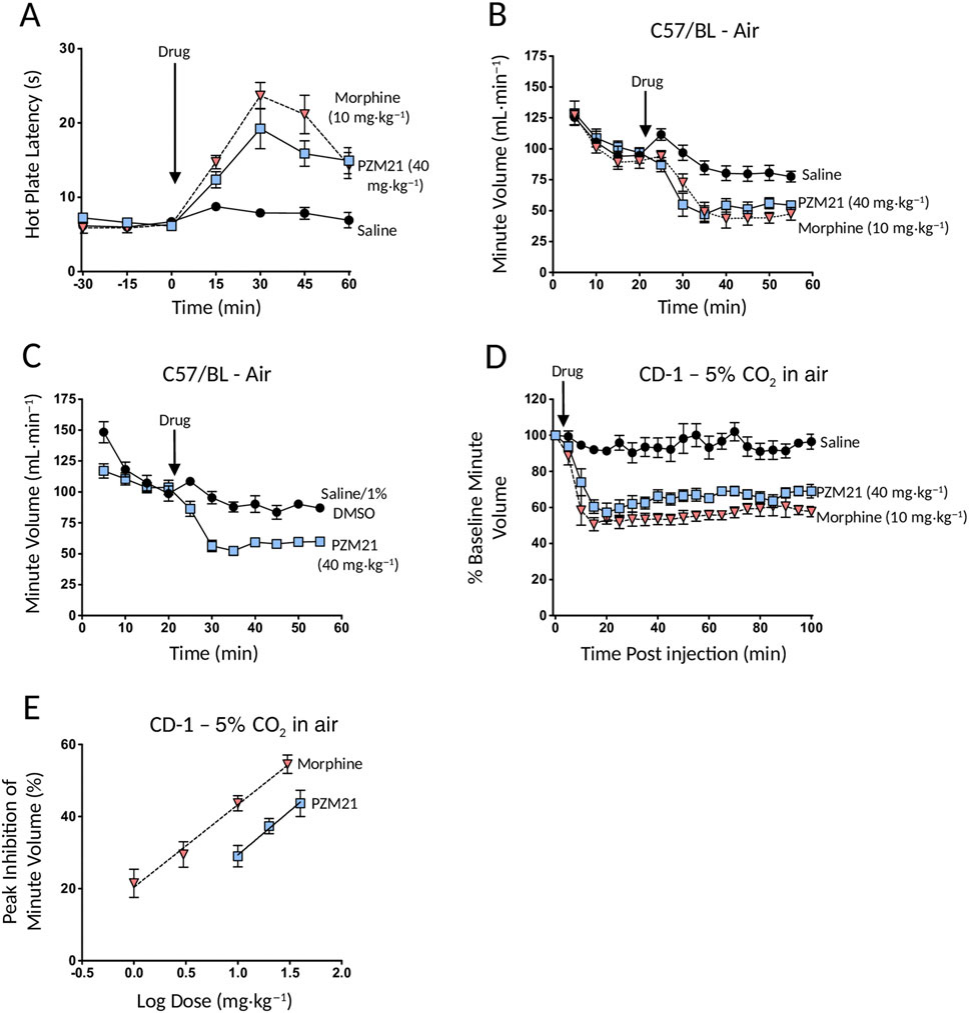
Antinociception and respiratory depression induced by PZM21 and morphine in mice. (A) PZM21 (40 mg·kg^−1^ i.p.) and morphine (10 mg·kg^−1^ i.p.) enhanced paw withdrawal latency in the mouse (CD‐1) hot plate antinociception test [withdrawal latencies for both PZM21‐ and morphine‐treated mice were statistically different from those in saline‐treated mice at all time points following drug injection; F = 43.53 (dfn = 2, dfd = 147) *P* < 0.05]. (B) PZM21 (40 mg·kg^−1^ s.c.) and morphine (10 mg·kg^−1^ s.c.) depressed respiration, measured as MV, in C57/BL mice breathing air. (C) A separately synthesized sample of PZM21 (40 mg·kg^−1^ i.p.) supplied by Dr Peter Gmeiner also depressed respiration in C57/BL mice breathing air. (D) PZM21 (40 mg·kg^−1^ i.p.) and morphine (10 mg·kg^−1^ i.p.) depressed respiration in CD‐1 mice breathing 5% CO_2_ in air for a period of 100 min post drug injection. In (B), the extent of respiratory depression induced by either PZM21 or morphine was statistically different from saline‐treated mice at 5 – 45 min post drug injection [F = 36.77 (dfn = 2, dfd = 165) *P* < 0.05] whereas in (C), statistical difference was observed at all time points 10 min post drug injection [F = 115.88 (dfn = 1, dfd = 110) *P* < 0.05] (asterisks to denote statistical significance are omitted from the figure to maintain clarity). In (D), the extent of respiratory depression induced by either PZM21 or morphine was statistically different from saline‐treated mice at 10–100 min time points following drug injection [F = 418.15 (dfn = 2, dfd = 315) *P* < 0.05]. (E) PZM21 (10–40 mg·kg^−1^ i.p.) and morphine (1–30 mg·kg^−1^ i.p.) depressed respiration in CD‐1 mice breathing 5% CO_2_ in air in a dose‐dependent manner. Percent respiratory depression has been calculated for each mouse at the peak effect of the drugs which for all doses occurred at 10 or 15 min after injection. In (A–E), data shown are means ± SEM (*N* = 6 for all groups). Data were analysed by two‐way ANOVA with Bonferroni's post test.

In the mouse (CD‐1) hot plate assay PZM21 (40 mg·kg^−1^ i.p.) increased latency for nociceptive behaviour for the duration of the 60 min observation period following drug administration (Figure 2A). The peak antinociceptive response induced by PZM21 (40 mg·kg^−1^ i.p.) was similar to that induced by morphine (10 mg·kg^−1^ i.p.) when compared by two‐way ANOVA with Bonferroni's comparison [F = 1.65 (dfn = 1, dfd = 98) *P* > 0.05] (Figures 2A and 5A).

In contrast to Manglik *et al*. (2016), we observed that in C57/BL mice breathing air PZM21 (40 mg·kg^−1^ injected s.c.) caused significant respiratory depression (Figure 2B) that was rapid in onset, the peak effect being attained 10 – 15 min after injecting the drug, and persisted for the duration of the 30 min post injection observation period. The decrease in MV caused by 40 mg·kg^−1^ PZM21 was similar in amplitude to that observed following administration of 10 mg·kg^−1^ morphine (Figure 2B). To confirm that the respiratory depressant effect of PZM21 that we had observed was not due to the presence of any impurity, we obtained a sample of PZM21 from Dr Peter Gmeiner who synthesized the PZM21 used in the Manglik *et al*. (2016) study; we observed that 40 mg·kg^−1^ (i.p.) of this sample of PZM21 also depressed respiration (Figure 2C).

In mice breathing air, respiration gradually declines with time (see data for saline or saline/1% DMSO‐treated mice in Figure 2B, C and for vehicle‐injected mice in figure 4g in Manglik *et al*., 2016); animals also sometimes curl up ‘asleep’ which interferes with plethysmograph recording. To circumvent this, we performed experiments in which mice breathe 5% CO_2_ in air (see Hill *et al.,* 2016; Withey *et al.,* 2017). This increases MV, which in saline‐treated mice remains constant for periods of up to 90 min (Figure 2D). In CD‐1 mice breathing 5% CO_2_ in air, the respiratory depression induced by PZM21 (10 – 40 mg·kg^−1^ i.p.) was dose‐dependent and was maintained over the 60 min period following drug administration (Figure 2D, E). Over the range of doses tested, PZM21 was approximately fourfold less potent than morphine (Figure 2E).

**Figure 3:**
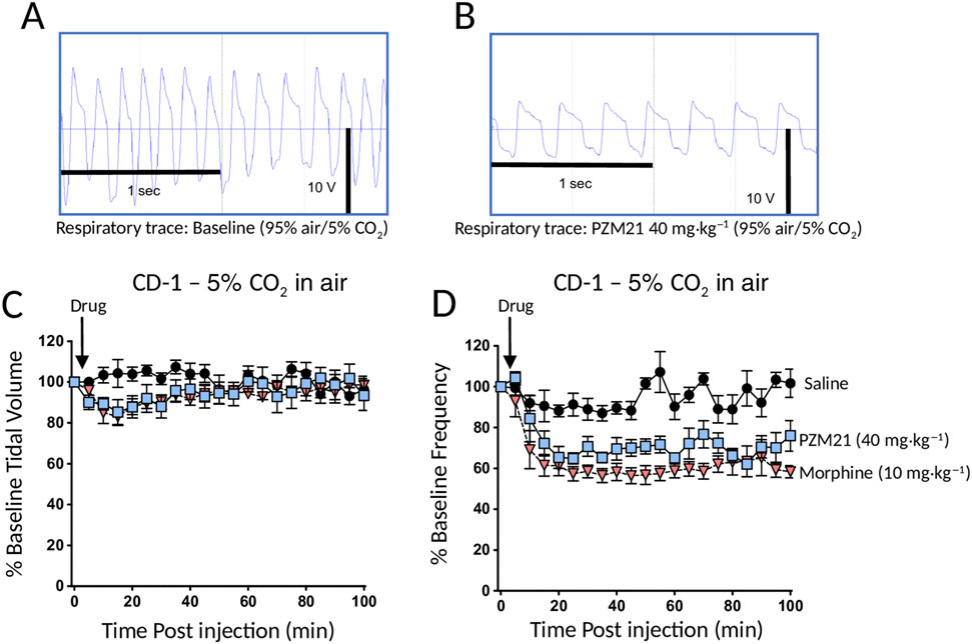
PZM21 and morphine depress respiratory rate not tidal volume. (A) and (B) show raw respiration traces recorded from a single CD‐1 mouse breathing 5% CO_2_ in air before (A) and after (B) administration of PZM21 (40 mg·kg^−1^ i.p.). The horizontal line indicates the point of pressure inflexion. On the respiration traces, inspiration is downwards. (C, D) In mice breathing 5% CO_2_ in air, PZM21 (40 mg·kg^−1^ i.p.) and morphine (10 mg·kg^−1^ i.p.) have no effect on tidal volume [F = 15.37 (dfn = 2, dfd = 315) *P* > 0.05] but reduce the rate of respiration at all time points following drug injection (morphine) and reduce the rate of respiration at 25 – 70, 85 and 95 – 100 min (PZM21) [F = 187,27 (dfn = 2, dfd = 110) *P* < 0.05]. Data shown are means ± SEM (*N* = 6 in all groups) and were analysed by two‐way ANOVA with Bonferroni's post test.

The decrease in MV in response to PZM21 or morphine resulted from a decrease in respiratory rate rather than a decrease in tidal volume (Figure 3). Although the depth of respiration was reduced, tidal volume was maintained because the duration of inspiration was prolonged by an apneustic compensation (see also Hill *et al.,* 2016).

**Figure 4:**
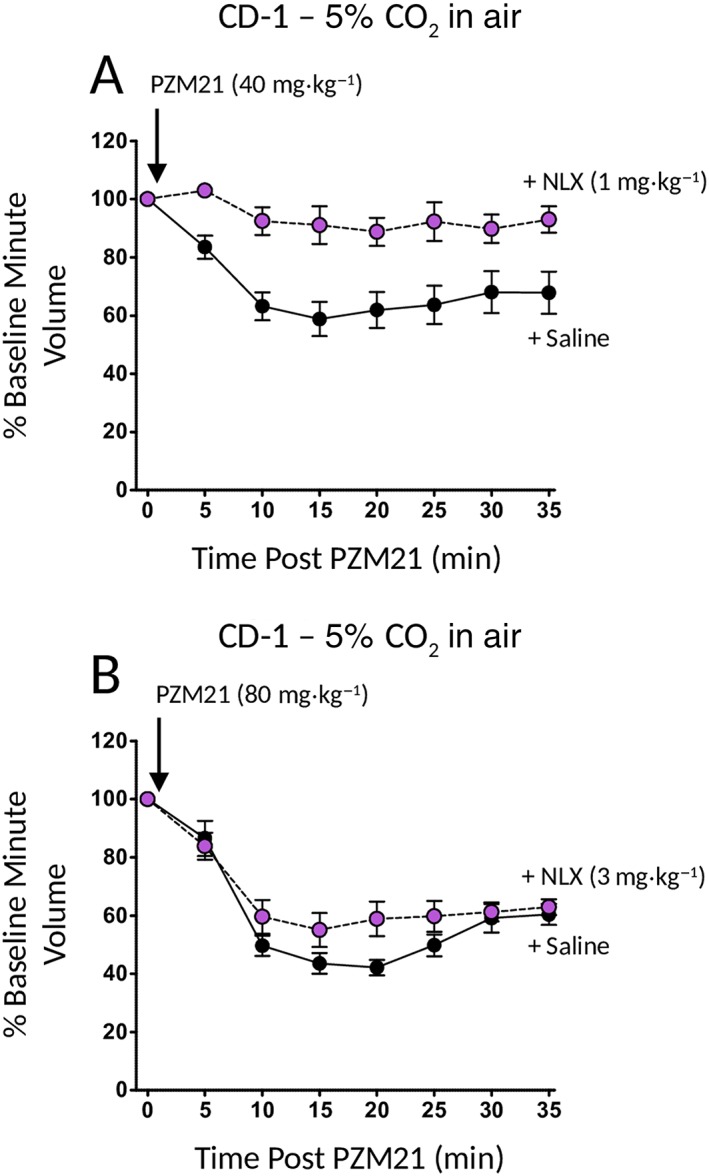
Naloxone reversal of respiratory depression induced by PZM21. (A) Treatment with naloxone (1 mg·kg^−1^ i.p.; NLX) at the same time as PZM21 injection prevented the respiratory depression in CD‐1 mice induced by PZM21 (40 mg·kg^−1^). Respiratory depression following PZM21 injection was significantly less in naloxone‐treated mice than in saline‐treated mice at all time points following drug injection [F = 76.63 (dfn = 1, dfd = 80) *P* < 0.05]. (B) In contrast, the respiratory depression induced by PZM21 (80 mg·kg^−1^ i.p.) in CD‐1 mice was not reversed by a higher dose of naloxone (3 mg·kg^−1^ i.p.) [F = 8.58 (dfn = 1, dfd = 110) *P* > 0.05]. Data shown are means ± SEM (*N* = 6 in all groups) and were analysed by two‐way ANOVA with Bonferroni's post test.

Administration of naloxone (1 mg·kg^−1^ i.p.) along with PZM21 (40 mg·kg^−1^ i.p.) prevented the respiratory depression induced by PZM21 (Figure 4A), demonstrating that this effect of PZM21 was mediated by opioid receptor activation. However, when the dose of PZM21 was increased to 80 mg·kg^−1^ (i.p.), the degree of respiratory depression also increased, but in this instance, the effect of PZM21 was not fully reversed even by a higher dose of naloxone (3 mg·kg^−1^ i.p.) (Figure 4B). This indicates that at high doses, the respiratory depression by PZM21 may involve a non‐opioid receptor‐mediated component.

### Differential development of tolerance to antinociception and respiratory depression by PZM21

We have previously used a twice daily injection of opioid to study the development of tolerance to the antinociceptive (tail flick test) and respiratory depressant effects of morphine and observed that tolerance developed to the antinociceptive action of morphine but not to the respiratory depressant action (Hill *et al.,* 2016). Here, we utilized a similar twice daily opioid injection protocol in which mice were injected at 09:00 and 21:00 h each day with PZM21 (40 mg·kg^−1^ i.p.) or morphine (10 mg·kg^−1^ i.p.) for up to 5 days.

**Figure 5:**
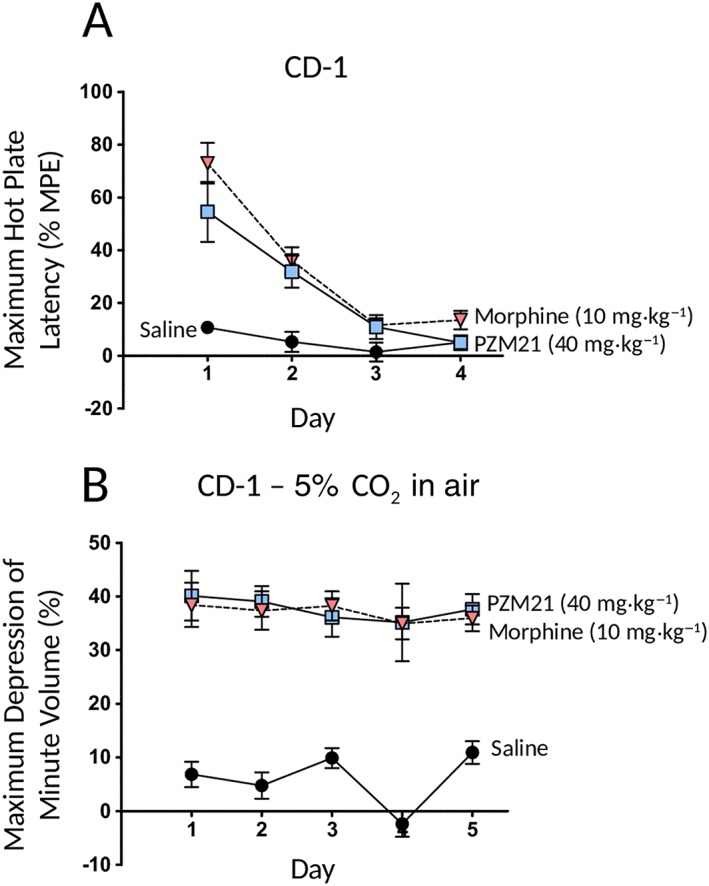
Repeated injection of PZM21 or morphine induced tolerance to their antinociceptive effect but not to their respiratory depressant effect. (A). Antinociceptive responses to the first injection of PZM21 (40 mg·kg^−1^), morphine (10 mg·kg^−1^) or saline on each day in mice (CD‐1) receiving twice daily doses of either drug for 4 days. Data shown are means± SEM (*N* = 8 in all groups) of the maximum increase in hot plate latency (% MPE) after administering the drug. For both PZM21 and morphine, there was a progressive reduction in response on days 2–4 [F = 8.56 (dfn = 6, dfd = 63) *P* < 0.05]. Data in (A) have been analysed by two‐way repeated measures ANOVA. (B) Maximum respiratory depression in response to the first injection of PZM21 (40 mg·kg^−1^), morphine (10 mg·kg^−1^) or saline on each day in mice (CD‐1) receiving twice daily doses of drug for 5 days. Data shown are means± SEM (*N* = 6 in both groups). The data for morphine are recalculated from figure 2e of Hill *et al*. (2016). There was no reduction in the respiratory depressant effect of PZM21 or morphine over 5 days of drug administration [F = 0.83 (dfn = 8, dfd = 110) *P* > 0.05]. Data in (A) and (B) have been analysed by two‐way repeated measures ANOVA with Bonferroni's post test.

Repeated injection of PZM21 or morphine resulted in the development of tolerance to their antinociceptive effects as measured in the hot plate assay (Figure 5A). By the fourth day of treatment, PZM21 and morphine did not induce any antinociception. In a separate group of mice, we observed that tolerance to the respiratory depressant effect of PZM21 did not develop even after 5 days of treatment (Figure 5B). For comparison with morphine, data showing lack of tolerance development to respiratory depression with repeated morphine dosing have been recalculated from Hill *et al*. (2016) and included in Figure 5B.

## Discussion

The main finding of this study is that the structurally novel μ receptor agonist PZM21 induces marked respiratory depression, similar to morphine. This finding is at odds with a previous study where PZM21 was reported not to induce respiratory depression (Manglik *et al.,* 2016). In addition to our findings with the sample of PZM21 that we synthesized, we also obtained a sample of PZM21 from the original investigators and still observed respiratory depression with that sample. We observed respiratory depression in both C57BL and CD‐1 mice showing that the response is not strain specific. Furthermore, we observed respiratory depression when PZM21 was injected i.p. or s.c. and when animals breathed air or 5% CO_2_ in air to stabilize respiration across the observation period. We did not observe depression of respiration when mice were injected with vehicle alone. This is in contrast to Manglik *et al*. (2016) who observed a large effect in vehicle‐injected mice that may have obscured the respiratory depressant effect of PZM21.

We have also demonstrated that repeated administration of PZM21 induced tolerance to its antinociceptive effect, similar to that seen with morphine, but with this treatment schedule, PZM21 did not induce tolerance to respiratory depression, again as seen with morphine.

PZM21 is a low efficacy agonist for both G protein and arrestin signalling. The maximum response to PZM21 compared to that of DAMGO does appear to be lower for arrestin‐3 translocation than for G_i_ activation (compare Figures 1A, B). This could indicate that PZM21 exhibits some bias for G_i_ activation over arrestin‐3 translocation. However, with low efficacy agonists such as PZM21, the error in EC_50_ values obtained from the concentration–response curves is large (see Table 1) which makes conclusions about bias difficult. The problems of quantifying bias with low efficacy agonists and comparing bias between different cell types and assays are increasingly being acknowledged (Thompson *et al.,* 2015; Winpenny *et al.,* 2016; Michel and Charlton, 2018). It should be noted that in Manglik *et al*. (2016), the authors were unable to formally demonstrate that PZM21 was G protein‐biased, even in the presence of overexpressed http://www.guidetopharmacology.org/GRAC/ObjectDisplayForward?objectId=1466 to amplify the arrestin response.

In the present study, PZM21 did produce antinociception in the hot plate assay, but contrary to the previous report on this drug (Manglik *et al.,* 2016), we also observed that PZM21 produced prolonged respiratory depression. PZM21 is therefore very similar to the prototypic opioid morphine, although *in vivo* it is less potent. While the current dogma is that, for μ receptor agonists, respiratory depression results from activation of arrestin signalling (see Chan *et al*., 2017; Madariaga‐Mazón *et al.,* 2017; Roth *et al.,* 2017; Smith *et al.,* 2018), there is evidence that signalling through G_i_/G_o_ is also involved in mediating this behaviour. μ Receptor coupling through G_i_/G_o_ proteins results in activation of http://www.guidetopharmacology.org/GRAC/FamilyDisplayForward?familyId=74 (GIRKs) and inhibition of voltage‐gated calcium channels (North and Williams, 1985; Seward *et al.,* 1991), and it has been reported that the respiratory depression induced by either DAMGO, applied locally in the ventrolateral medulla, or http://www.guidetopharmacology.org/GRAC/LigandDisplayForward?ligandId=1626, administered systemically, is attenuated in GIRK2 subunit knockout mice (Montandon *et al.,* 2016). The effect of DAMGO on respiration was also reduced in wild‐type mice by local administration of http://www.guidetopharmacology.org/GRAC/LigandDisplayForward?ligandId=2383, a blocker of GIRK channels. This would imply that respiratory depression involves μ receptors coupling to GIRK channels through activation of G_i_/G_o_ proteins. Furthermore, the role of G_i_/G_o_‐coupling to voltage‐dependent calcium channels in respiratory depression has not been evaluated. On the other hand, morphine‐induced respiratory depression was reported to be reduced in arrestin‐3 knockout mice (Raehal *et al.,* 2005), while Schmid *et al*. (2017) have recently described several novel G protein‐biased μ receptor ligands that appear to induce less respiratory depression than morphine at equi‐antinociceptive doses. Nevertheless, our present results with PZM21 question the assumption that arrestin‐dependent signalling mediates the respiratory depression observed with μ receptor agonists.

We observed that on repeated administration, tolerance developed to the antinociceptive effect of PZM21, as has previously been widely reported with morphine (see references in Williams *et al.,* 2013). This finding suggests that the evasion of tolerance to the analgesic effects, a suggested benefit of G protein‐preferring μ receptor agonists (Smith *et al.,* 2018), is not achieved and that instead such agonists remain able to trigger signalling mechanisms that lead to profound tolerance. On the other hand, with twice daily injections tolerance did not develop to the respiratory depressant effect of PZM21. We have previously reported similar differential tolerance development to morphine (Hill *et al.,* 2016). With morphine, more sustained drug delivery using subcutaneous pellet or osmotic mini‐pump implantation did result in the development of tolerance to respiratory depression but at a slower rate than tolerance to antinociception (Hill *et al.,* 2016; Withey *et al.,* 2017). Unfortunately, we had insufficient supplies of PZM21 to perform further experiments with osmotic mini‐pump delivery. The molecular mechanisms underlying the development of tolerance to μ receptor agonists, particularly lower efficacy agonists that have lower propensity for arrestin recruitment, are complex and contentious (see Williams *et al.,* 2013). Future studies should address the mechanism(s) underlying tolerance induced by prolonged PZM21 treatment.

### Conclusions

In conclusion, our data on respiratory depression are in direct contrast to the previously published observation by Manglik *et al*. (2016) that PZM21 did not cause respiratory depression in mice. Given that PZM21 did induce some arrestin‐3 translocation as well as G_i_ activation, we are unable to conclude definitively that, for μ receptor agonists, respiratory depression is mediated by G protein or arrestin signalling. The future identification of higher efficacy μ receptor agonists showing greater bias for G protein over arrestin signalling remains a promising avenue for the development of novel opioid agonists and should answer this important question.

## Author contributions

R.H. performed the *in vivo* respiration and antinociception experiments, analysed the data and prepared the figures. A.D. and S.H. synthesized PZM21. A.C. and K.S. performed the *in vitro* BRET experiments and analysed the data. W.D., C.B., E.K. and G.H. conceived the study and wrote the paper. All authors revised the manuscript.

## Conflict of interest

The authors declare no conflicts of interest.

## Declaration of transparency and scientific rigour

This http://onlinelibrary.wiley.com/doi/10.1111/bph.13405/abstract acknowledges that this paper adheres to the principles for transparent reporting and scientific rigour of preclinical research recommended by funding agencies, publishers and other organisations engaged with supporting research.
